# Supplementary figures and images for: Development of a 5-mRNAsi-related gene signature to predict the prognosis of colon adenocarcinoma
Source: PeerJ. 2023 Nov 24;11:e16477. doi: 10.7717/peerj.16477 (PMC10680455; doi:10.7717/peerj.16477)

**A**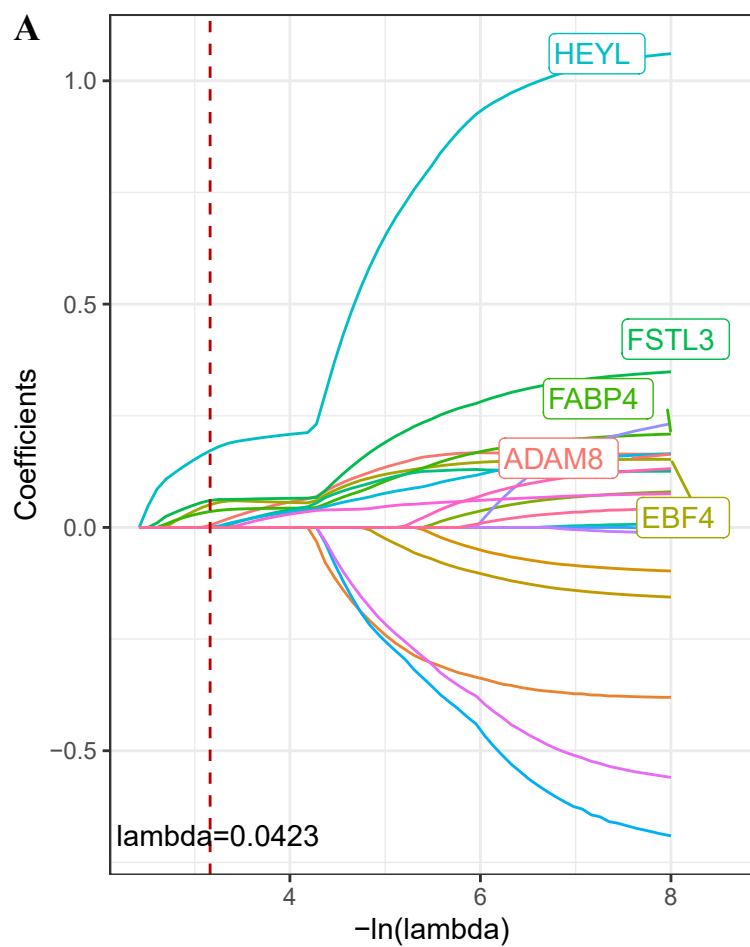**B**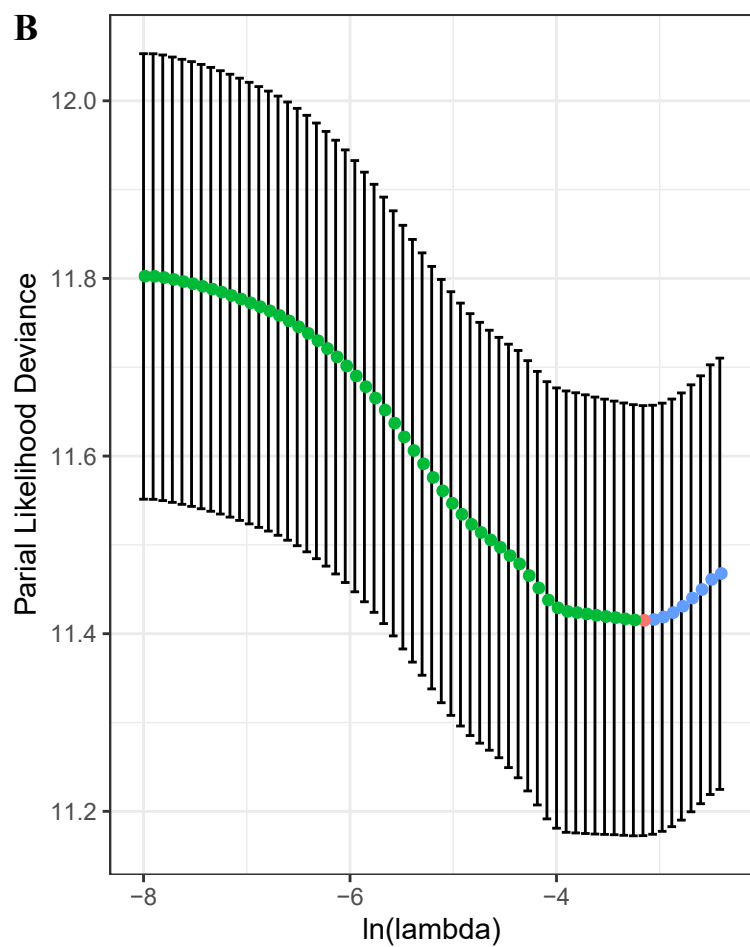

Supplement: Supplemental Information 1 — LASSO analysis in glmnet package showed trajectory and confidence interval of lambda. [file peerj-11-16477-s001.pdf]
