# Supplementary material for: Development of a 5-mRNAsi-related gene signature to predict the prognosis of colon adenocarcinoma
Source: PeerJ. 2023 Nov 24;11:e16477. doi: 10.7717/peerj.16477 (PMC10680455; doi:10.7717/peerj.16477)
Supplement: Supplemental Information 2 [file peerj-11-16477-s002.pdf]

HEYL

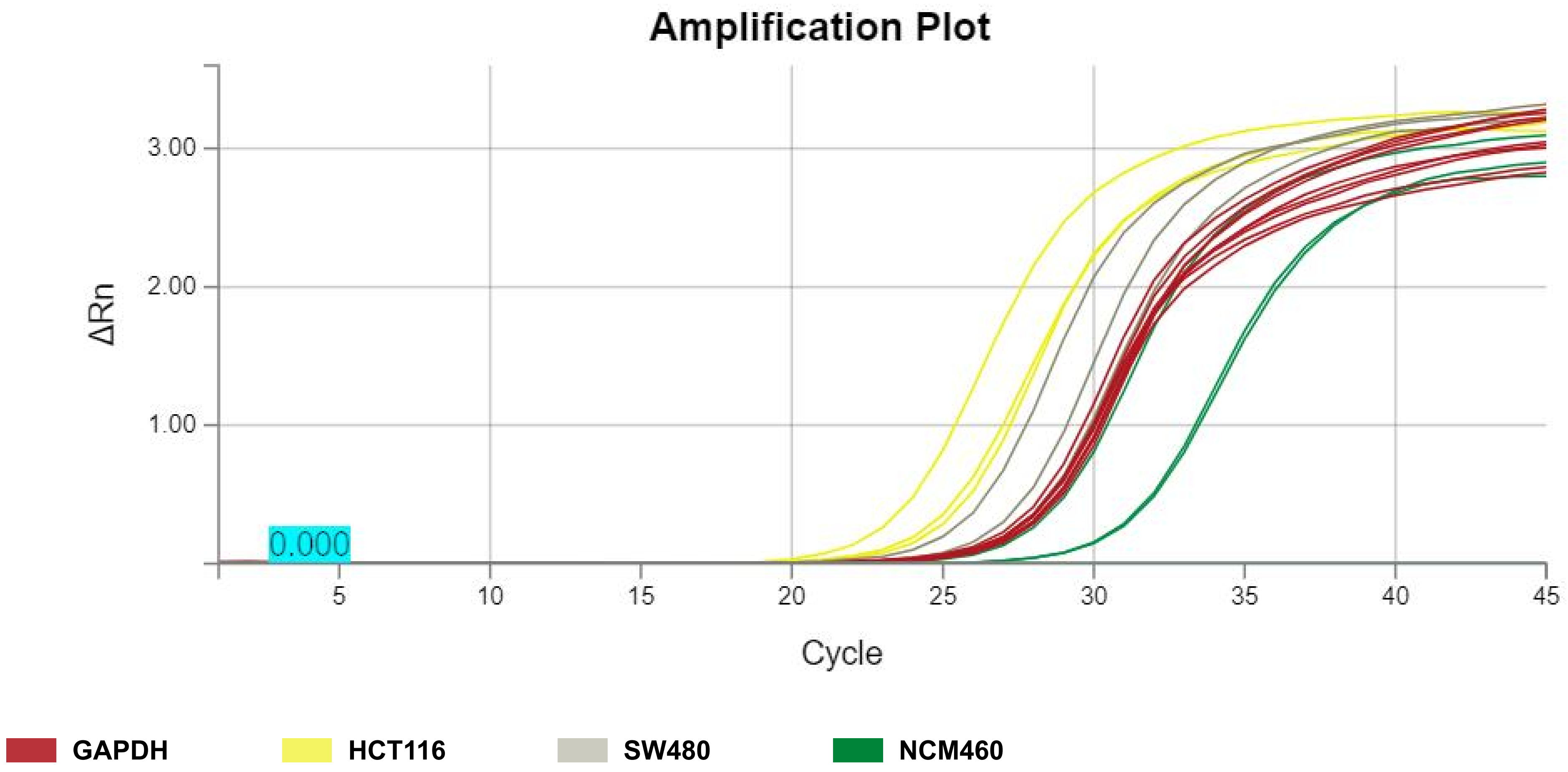
Amplification Plot
3.00
2.00
1.00
0.000
ΔRn
5
10
15
20
25
30
35
40
45
Cycle
GAPDH
HCT116
SW480
NCM460

HEYL

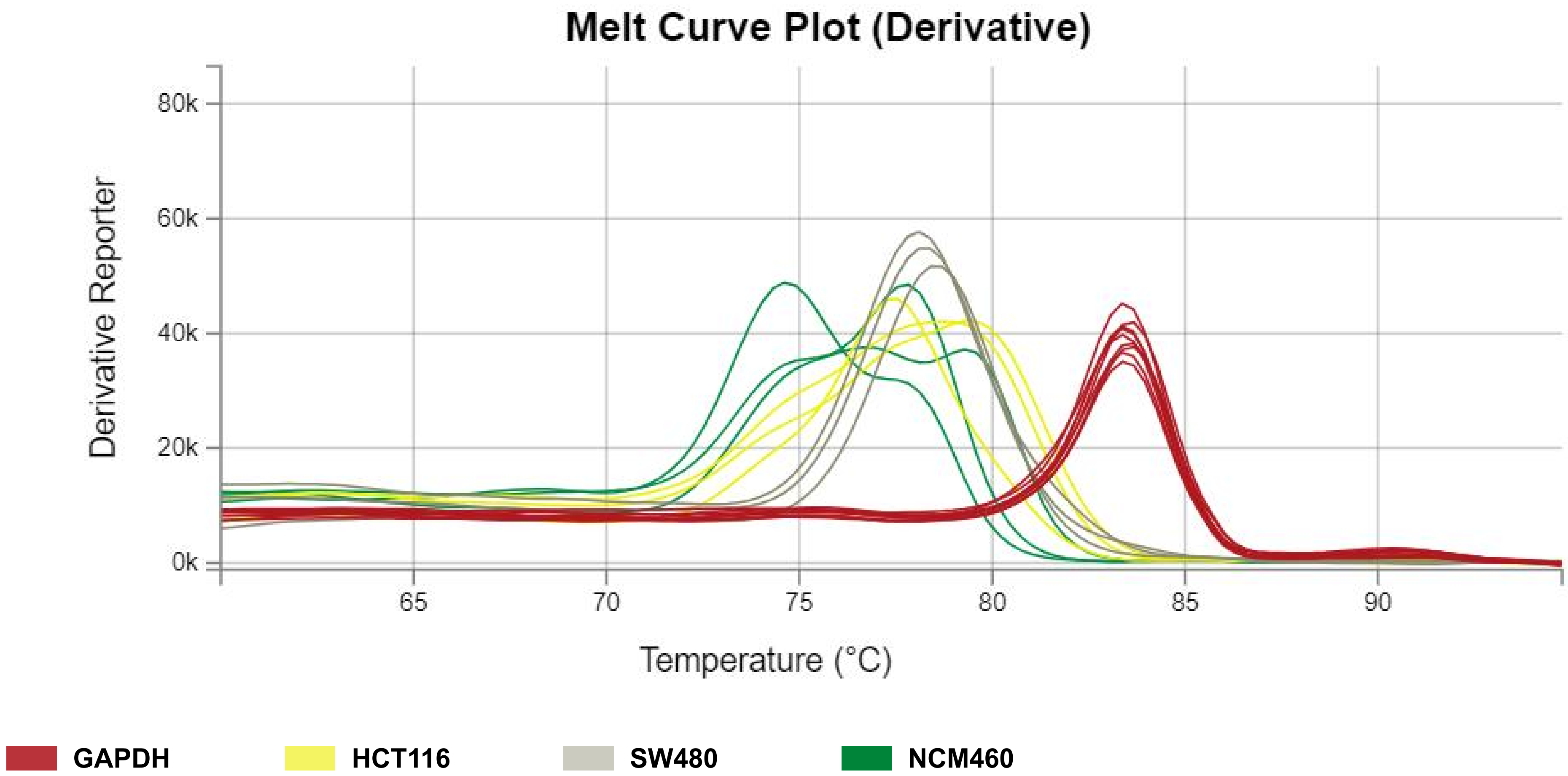
Melt Curve Plot (Derivative)
Derivative Reporter
80k
60k
40k
20k
0k
65
70
75
80
85
90
Temperature (°C)
GAPDH
HCT116
SW480
NCM460

FSTL3

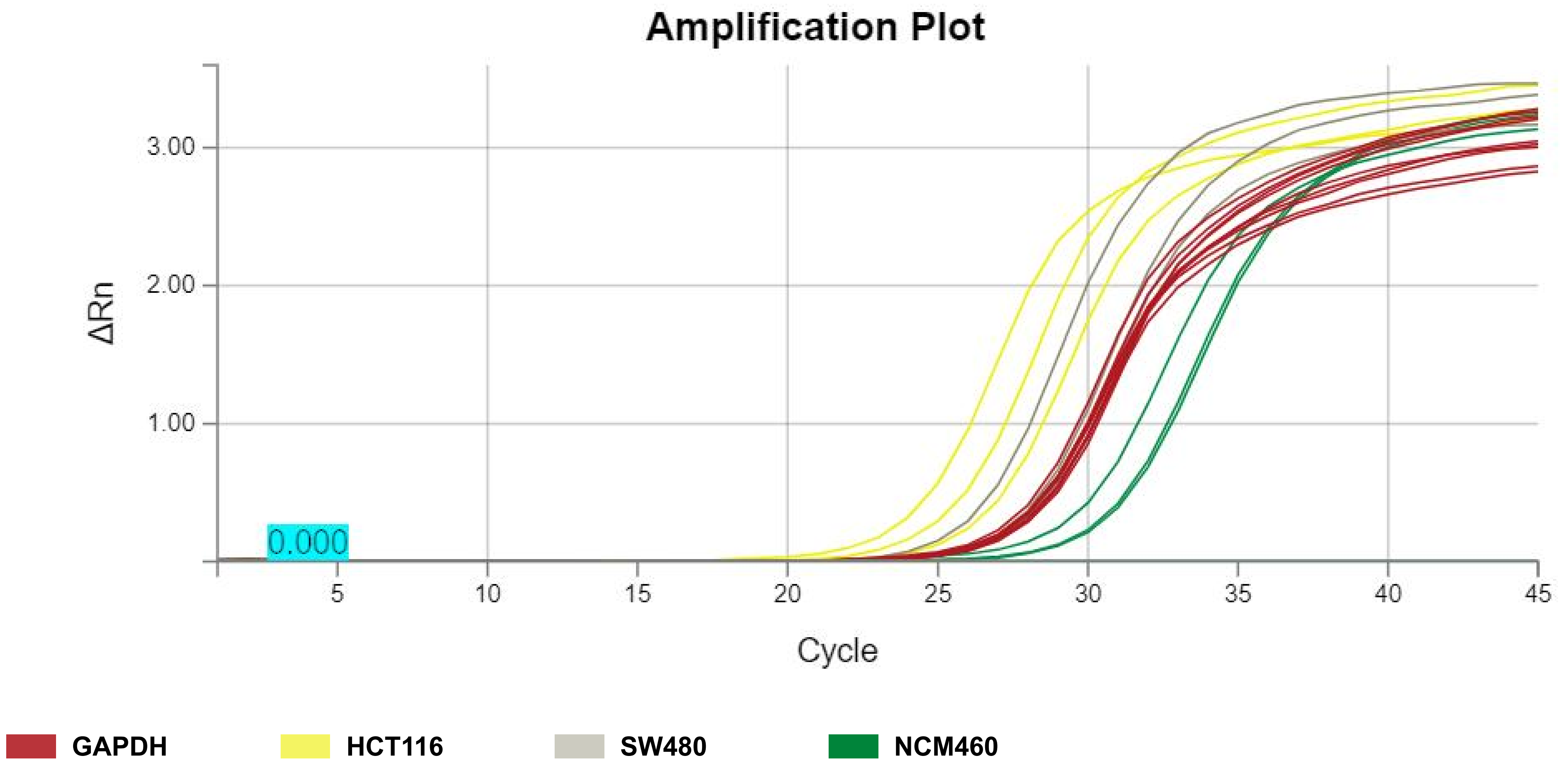

Amplification Plot
3.00
2.00
1.00
0.000
ΔRn
5
10
15
20
25
30
35
40
45
Cycle
GAPDH
HCT116
SW480
NCM460

FSTL3

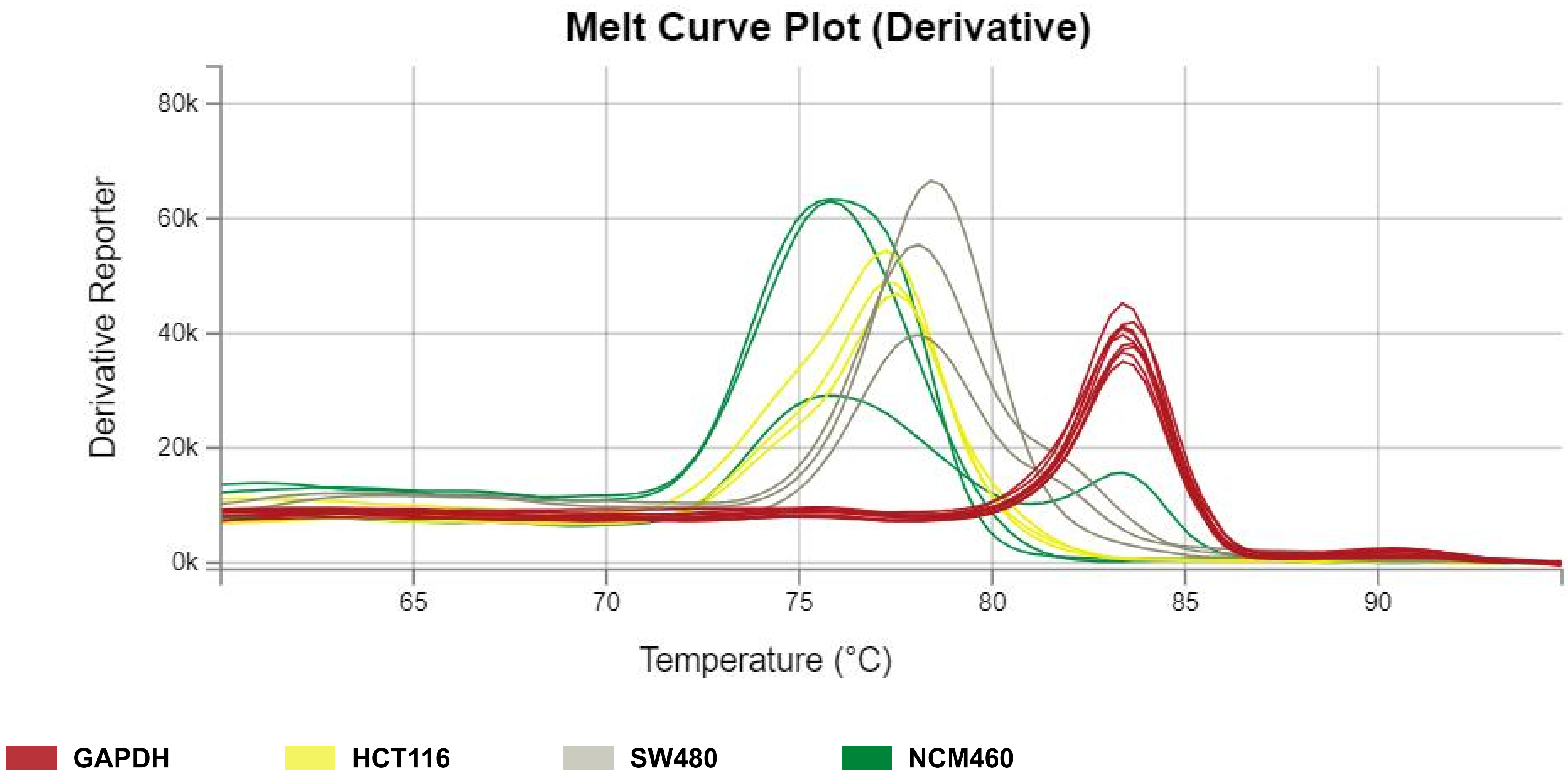
Melt Curve Plot (Derivative)
Derivative Reporter
80k
60k
40k
20k
0k
65
70
75
80
85
90
Temperature (°C)
GAPDH
HCT116
SW480
NCM460

FABP4

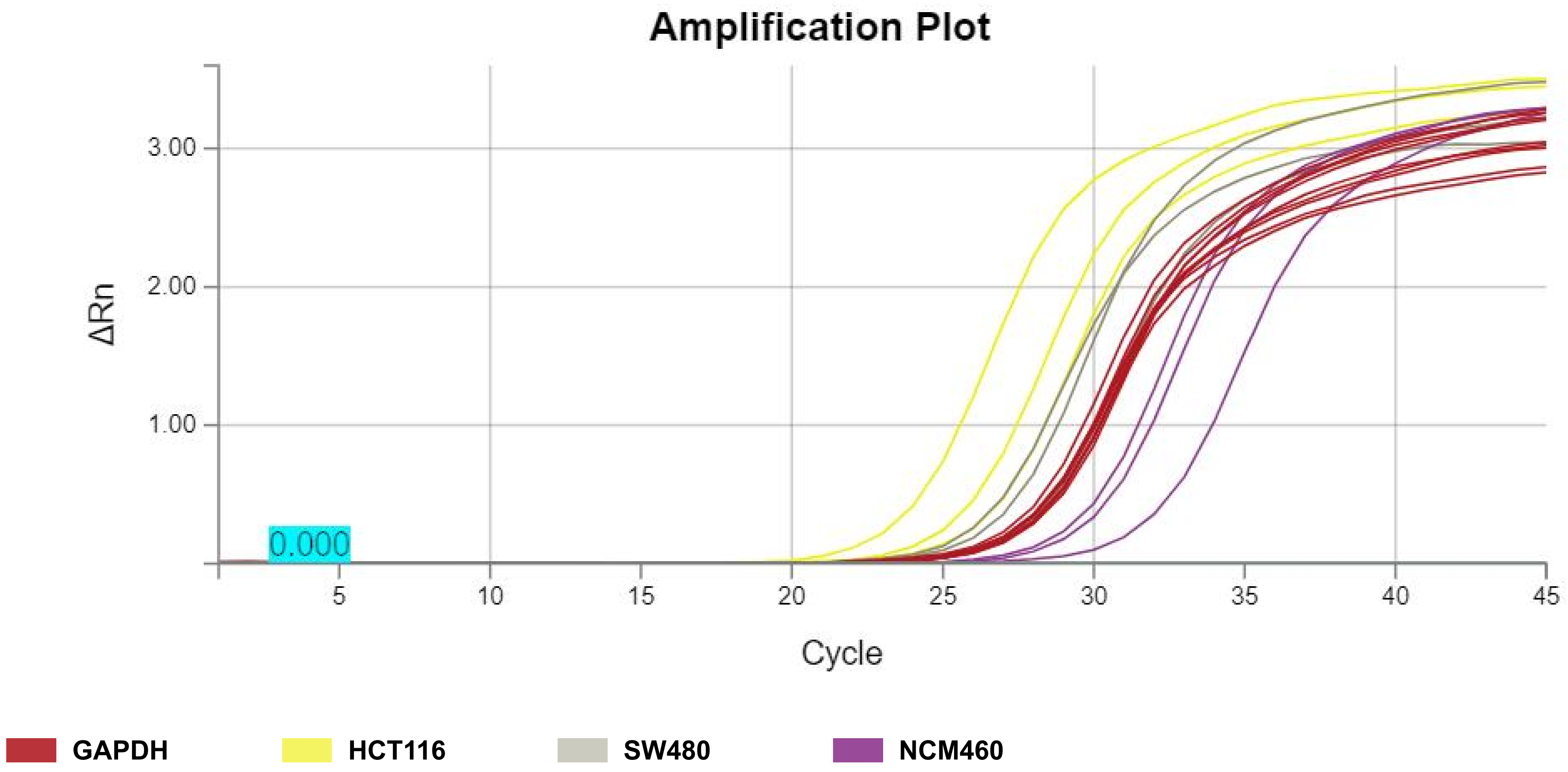

Amplification Plot
ΔRn
3.00
2.00
1.00
0.000
5
10
15
20
25
30
35
40
45
Cycle
GAPDH
HCT116
SW480
NCM460

FABP4

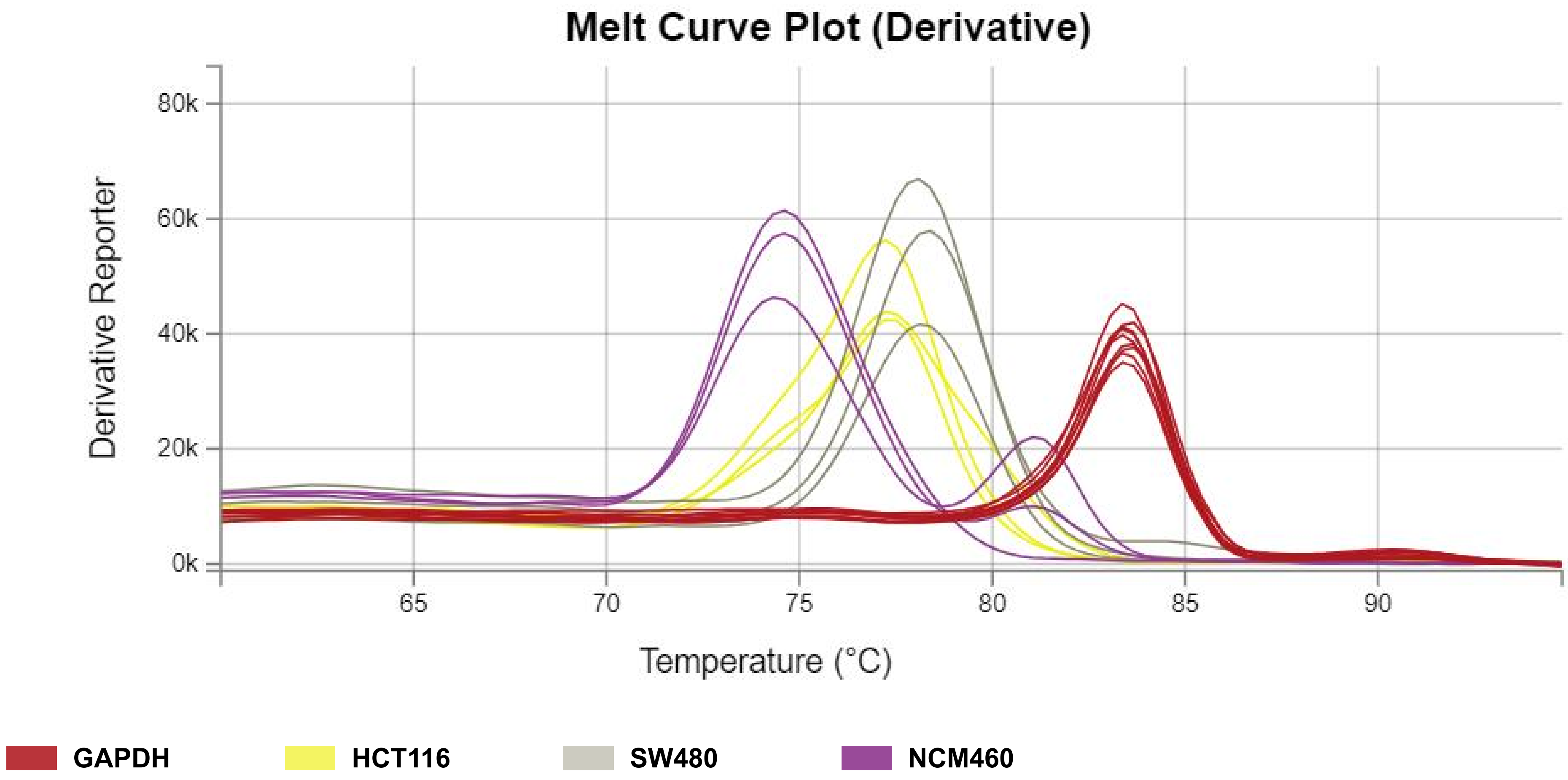
Melt Curve Plot (Derivative)
Derivative Reporter
80k
60k
40k
20k
0k
65
70
75
80
85
90
Temperature (°C)
GAPDH
HCT116
SW480
NCM460

ADAM8

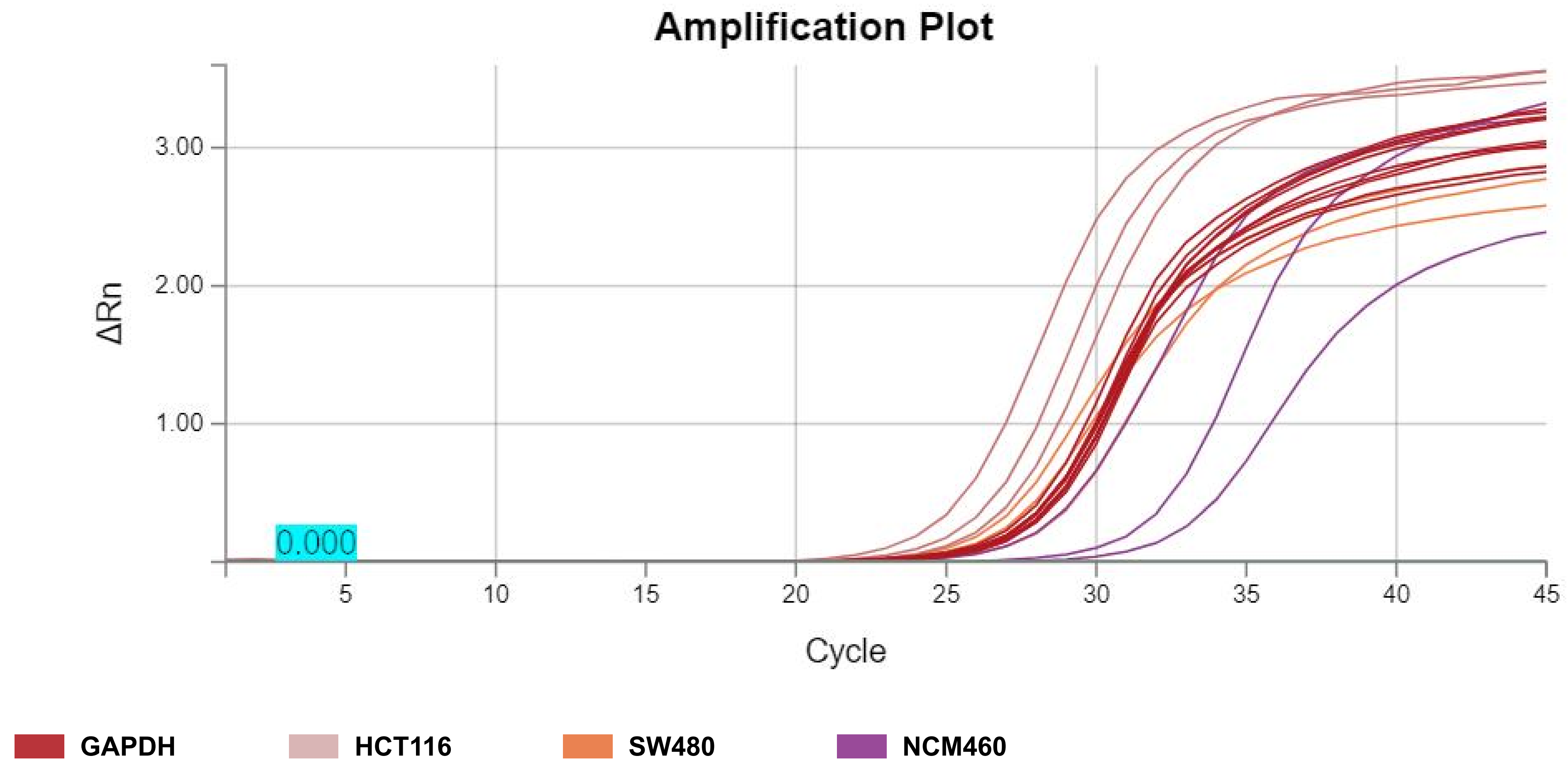
Amplification Plot
3.00
2.00
1.00
ΔRn
0.000
5
10
15
20
25
30
35
40
45
Cycle
GAPDH
HCT116
SW480
NCM460

ADAM8

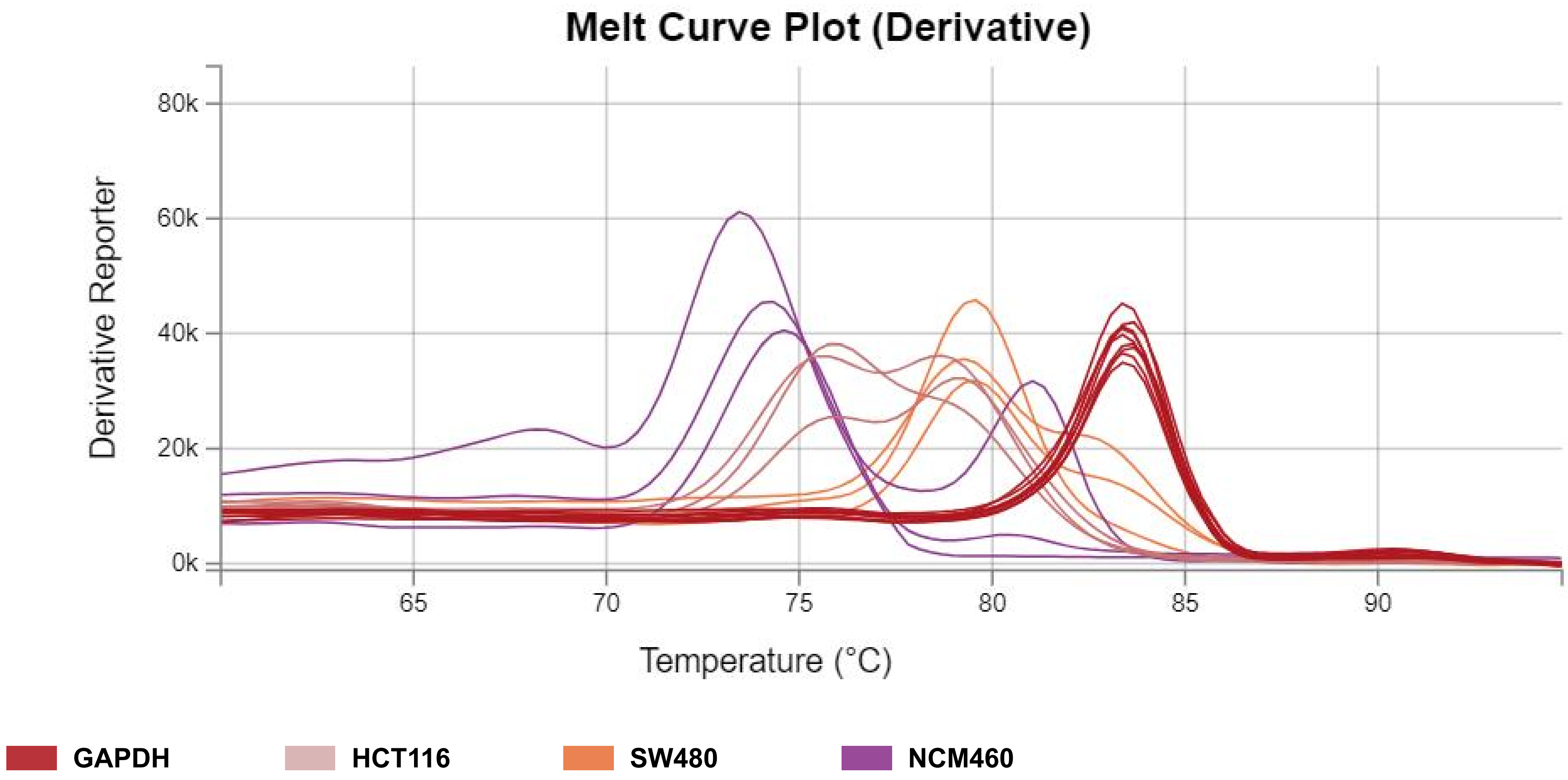

Melt Curve Plot (Derivative)
Derivative Reporter
80k
60k
40k
20k
0k
65
70
75
80
85
90
Temperature (°C)
GAPDH
HCT116
SW480
NCM460

EBF4

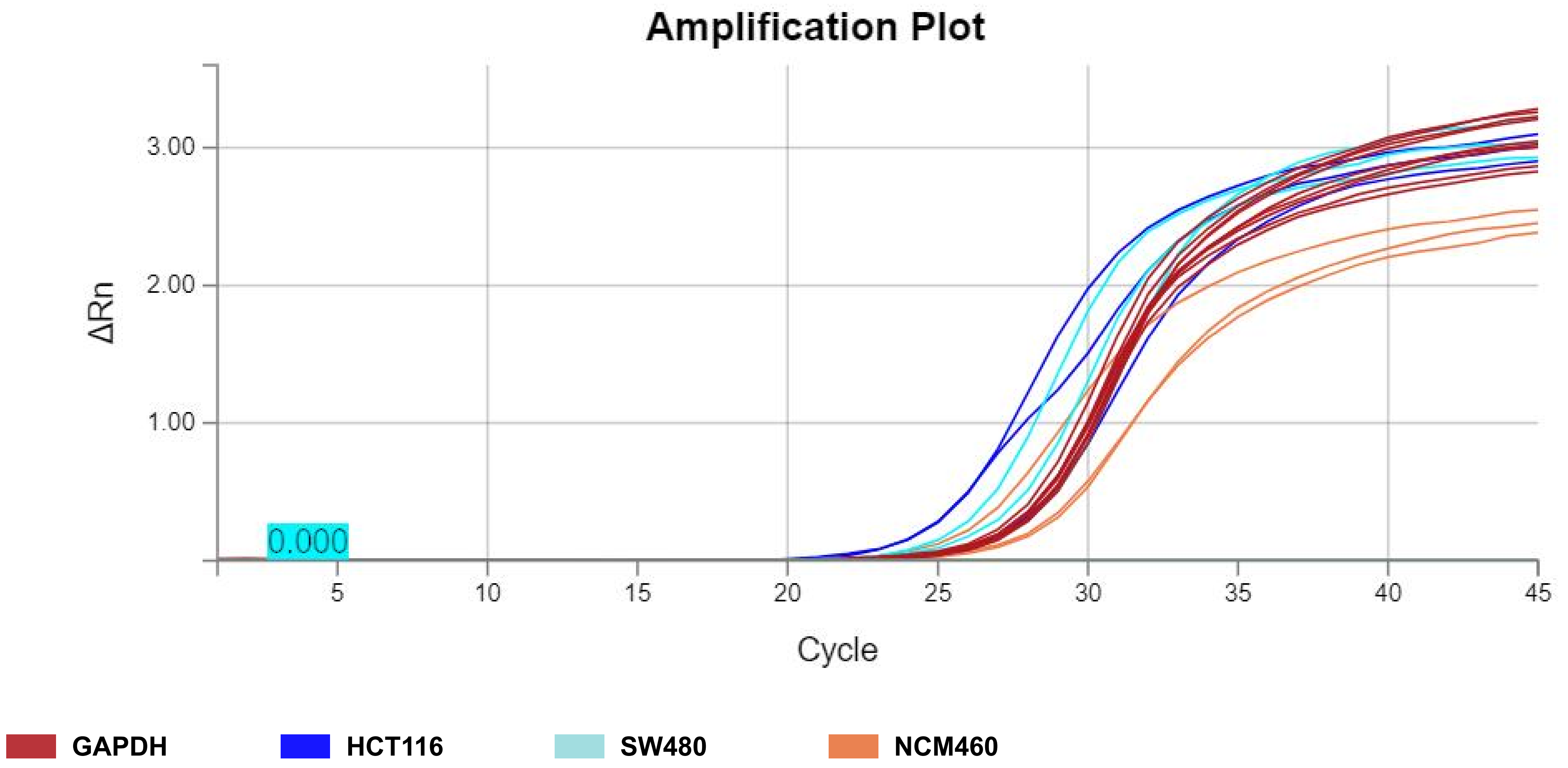

Amplification Plot
ΔRn
3.00
2.00
1.00
0.000
5
10
15
20
25
30
35
40
45
Cycle
GAPDH
HCT116
SW480
NCM460

EBF4

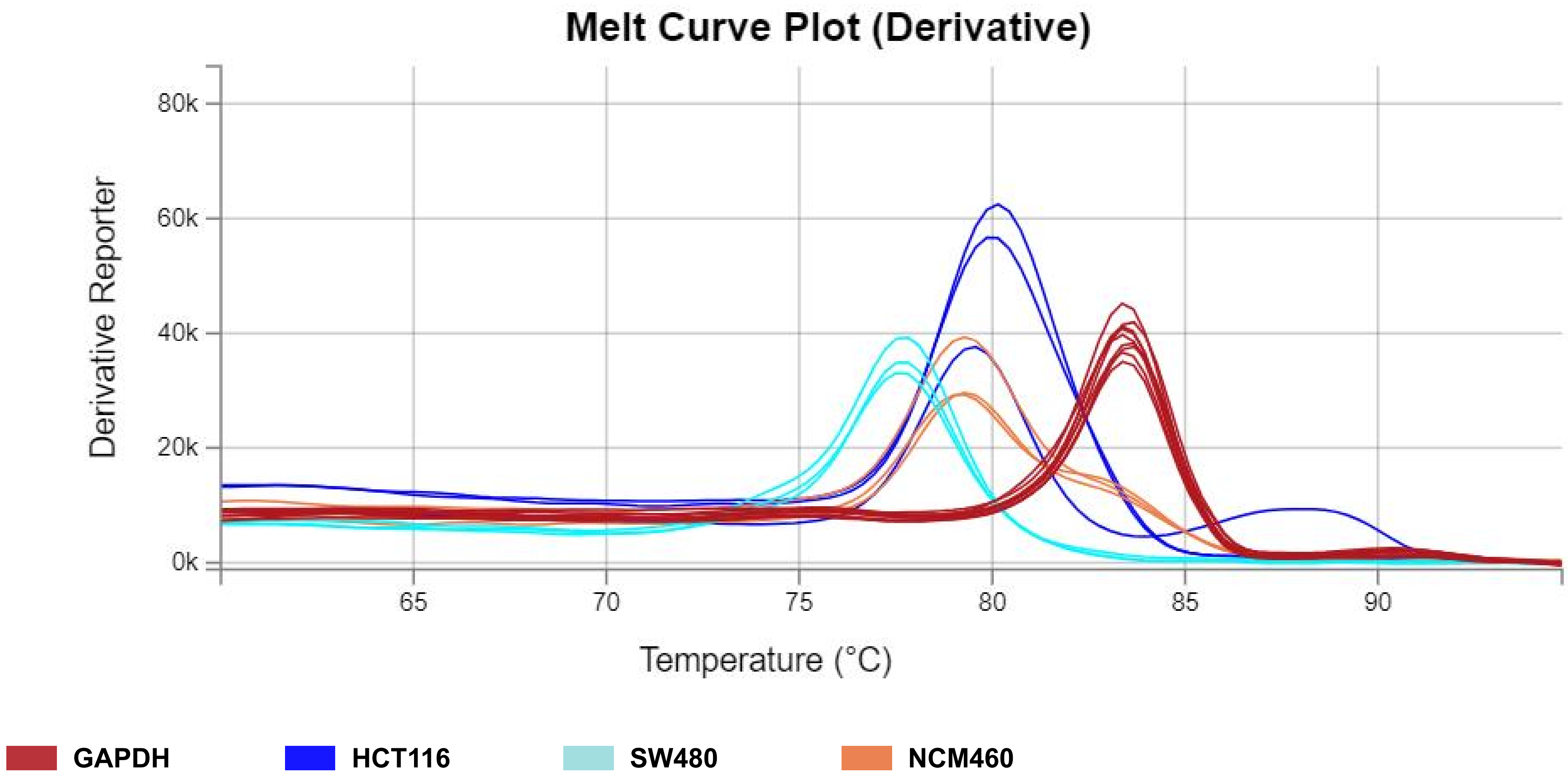
Melt Curve Plot (Derivative)
Derivative Reporter
80k
60k
40k
20k
0k
65
70
75
80
85
90
Temperature (°C)
GAPDH
HCT116
SW480
NCM460
