# Supplementary material for: Development of a 5-mRNAsi-related gene signature to predict the prognosis of colon adenocarcinoma
Source: PeerJ. 2023 Nov 24;11:e16477. doi: 10.7717/peerj.16477 (PMC10680455; doi:10.7717/peerj.16477)
Supplement: Supplemental Information 4 [file peerj-11-16477-s004.docx]

**MIQE Checklist**

**EXPERIMENTAL DESIGN**

Definition of experimental and control groups

In this experiment, the control group was defined as human breast normal epithelial cells NCM460, while the experimental group was defined as HCT116 and SW480 human breast cancer cell lines.

Number within each group

The number of each group was 3 independent experiments.

**SAMPLE**

Description

Microdissection or microdissection

We did not use techniques such as microdissection or microdissection to obtain samples.

Processing procedure

If frozen - how and how quickly?

We resuspended the NCM460, HCT116 and SW480 cell lines using serum-free cell cryopreservative and immediately froze them in a -80°C refrigerator.

If fixed - with what, how quickly?

No fix.

Sample storage conditions and duration (especially for FFPE samples

Samples were frozen with -80°C refrigerator and stored for one week.

**NUCLEIC ACID EXTRACTION**

Procedure and/or instrumentation

Name of kit and details of any modifications

TRIzol reagent (15596026, ThermoFisher, USA); DEPC water (BL510A, Biosharp, China); chloroform (T819286, MACKLIN, China); isopropanol (I811932, MACKLIN, China); anhydrous ethanol (E809056, MACKLIN, China).

Details of DNase or RNAse treatment

The consumables we use are RNAase and DNAase free. Prior to RNA extraction, all consumables were immersed in DEPC water overnight and used after sterilization.

Contamination assessment (DNA or RNA)

Contamination assessment is not performed by the lab technician.

Nucleic acid quantification

Instrument and method

After resuspension of RNA, evaluation of RNA concentration and treatment was performed. We used NanoDrop (ThermoFisher, USA) for RNA concentration and quality assessment. The RNA concentration should be above 500ng/ml and the A260/A280 ratio should be 1.8-2.0.

RNA integrity method/instrument

RIN/RQI or Cq of 3' and 5' transcripts

We used agarose gel electrophoresis for RNA integrity experiments. The results showed good RNA integrity.

Inhibition testing (Cq dilutions, spike or other)

We did not perform inhibition testing.

**REVERSE TRANSCRIPTION**

Complete reaction conditions

Amount of RNA and reaction volume

We used the High-Capacity cDNA Reverse Transcription Kit (4368814, ThermoFisher, USA) for reverse transcription. RNA volume: 5ul; Reaction volume: 20ul.

Priming oligonucleotide (if using GSP) and concentration

The concentration of priming oligonucleotides was 1ul in total 20ul volume.

Reverse transcriptase and concentration

The concentration of reverse transcriptase was 1ul in total 20ul volume.

Temperature and time

42℃: 60min; 72℃: 5min; 4℃: Save.

**qPCR TARGET INFORMATION**

If multiplex, efficiency and LOD of each assay.

No multiplex.

Sequence accession number

HEYL: NM_014571;

FSTL3: NM_005860;

FABP4: NM_001442;

ADAM8: NM_001109;

EBF4: NM_001110514.

Location of each primer by exon or intron (if applicable)

No applicable.

What splice variants are targeted?

No applicable.

**qPCR OLIGONUCLEOTIDES**

Primer sequences

| Gene | Forward primer sequence (5’-3’) | Reverse primer sequence (5’-3’) |
| --- | --- | --- |
| HEYL | ATGAAGCGACCCAAGGAGCC | GGCTACTGTTGATGCGGTCT |
| FSTL3 | GTGCCTCCGGCAACATTGA | GCACGAATCTTTGCAGGGA |
| FABP4 | ACTGGGCCAGGAATTTGACG | CTCGTGGAAGTGACGCCTT |
| ADAM8 | GAGGGTGAGCTACGTCCTTG | CAGCCGTATAGGTCTCTGTGT |
| EBF4 | TTCGTGGAAAAGGACCGAGAG | GGCACATTTCGGGGTTCTTG |
| GAPDH | AATGGGCAGCCGTTAGGAAA | GCCCAATACGACCAAATCAGAG |

Location and identity of any modifications

No modifications.

**qPCR PROTOCOL**

Complete reaction conditions

Reaction volume and amount of cDNA/DNA

Reaction volume: 20ul; cDNA amount: 2ul.

Primer, (probe), Mg++ and dNTP concentrations

Sense primer: 0.5ul; antisense primer: 0.5ul ; Mg++ concentration : 25nm/ml ; dNTP concentrations : 100umol/l.

Polymerase identity and concentration

Polymerase identity: RevertAidRNAse Reverse Ensyme (200);

Polymerase concentration: 1ul.

Temperature and time

Buffer/kit identity and manufacturer: High-Capacity cDNA Reverse Transcription Kit (4368814, ThermoFisher, USA).

Additives (SYBR Green I, DMSO, etc.)

FastStart Universal SYBR ®Green Master (Roche)

Complete thermocycling parameters

Cycling conditions started with an initial DNA denaturation phase at 95°C for 30 seconds, followed by 45 cycles at 94°C for 15 seconds, 56°C for 30 seconds, and 72°C for 20 seconds.

Manufacturer of qPCR instrument

LightCycler 480 PCR System (Roche)

**qPCR VALIDATION**

Specificity (gel, sequence, melt, or digest)


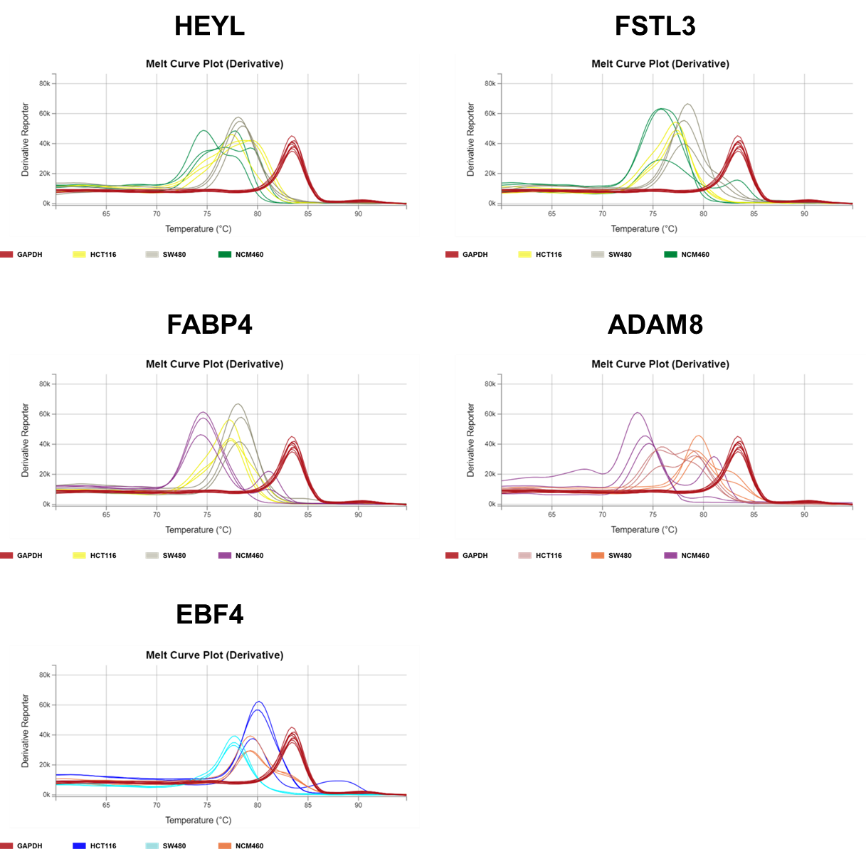


For SYBR Green I, Cq of the NTC

Cq: 44 cycle.

Standard curves with slope and y-intercept

PCR efficiency calculated from slope

No calculation.

Linear dynamic range

Linear dynamic range is in the acceptable range.

**DATA ANALYSIS**

qPCR analysis program (source, version)

ThermoFisher Design and analysis software 2.6.0.

Cq method determination

We take the start of the amplification curve as the Cq value.

Outlier identification and disposition

We remove outliers.

Results of NTCs

We found no abnormalities in the NTCs.

Description of normalisation method.

We normalized the results by GAPDH. The Cq value of the corresponding GAPDH was subtracted from the Cq value of the sample set.

Number and concordance of biological replicates

Three separate analyses were performed on each sample.

Repeatability (intra-assay variation)

Repeatability is fine.

Statistical methods for result significance

Based on the 2^-ΔΔCT^ method, data from the threshold cycle (CT) were obtained and standardized to the levels of GAPDH in each sample.

Software (source, version)

Excel and Graphpad Prism 9.4.1.
